# Effect of Paternal Age on Reproductive Outcomes of In Vitro Fertilization

**DOI:** 10.1371/journal.pone.0135734

**Published:** 2015-09-09

**Authors:** Yixuan Wu, Xiangjin Kang, Haiyan Zheng, Haiying Liu, Jianqiao Liu

**Affiliations:** 1 Department of Reproductive Medicine, the Third Affiliated Hospital of Guangzhou Medical University, Guangzhou, Guangdong, China; 2 Key Laboratory of Reproductive Medicine of Guangdong Province, Guangdong, China; 3 Key Laboratory for Major Obstetric Diseases of Guangdong Province, Guangdong, China; 4 Key Laboratory of Reproduction and Genetics of Guangdong Higher Education Institutes, Guangdong, China; Peiking University Third Hospital, CHINA

## Abstract

Although the adverse effects of maternal aging on reproductive outcomes have been investigated widely, there is no consensus on the impact of paternal age. Therefore, we investigated the effect of paternal age on reproductive outcomes in a retrospective analysis of 9,991 in vitro fertilization (IVF) cycles performed at the Reproductive Medicine Center of the Third Affiliated Hospital of Guangzhou Medical University (China) between January 2007 and October 2013. Samples were grouped according to maternal age [<30 (3,327 cycles), 30–34 (4,587 cycles), and 35–38 (2,077 cycles)] and then subgrouped according to paternal age (<30, 30–32, 33–35, 36–38, 39–41, and ≥42). The groups did not differ in terms of fertilization rate, numbers of viable and high-quality embryos and miscarriage rate when controlling maternal age (*P* >0.05). Chi-squared analysis revealed that there were no differences in implantation and pregnancy rates among the different paternal age groups when maternal age was <30 and 35–38 years (*P* >0.05). However, implantation and pregnancy rates decreased with paternal age in the 31–34 y maternal age group (*P* <0.05). Our study indicates that paternal age has no impact on fertilization rate, embryo quality at the cleavage stage and miscarriage rate. For the 30–34 y maternal age group, the implantation rate decreased with increased paternal age, with the pregnancy rate in this group being significantly higher in the paternal <30 y and 30–32 y age groups, compared with those in the 36–38 y and 39–41 y groups.

## Introduction

The age of parenthood has been steadily increasing over recent decades. In 2014, the Chinese government implemented an exemption to the one-child policy allowing a second child for families in which one parent, rather than both, is an only-child. As a result, many older couples are deciding to have a second child. The adverse effects of maternal aging on reproductive outcomes have been investigated widely, revealing that the pregnancy rate declines with maternal age, while the rates of miscarriage and birth defects increase. In contrast, the impact of advanced paternal age is comparatively less well-studied and there is no current consensus on its impact on reproduction outcomes.

Previous studies showed that advanced paternal age has adverse effects on sperm volume, motility and morphology [1]. Despite some discrepancies, reports suggest that increasing paternal age is associated with a higher incidence of aneuploidies [2]. Furthermore, the time to pregnancy also increases with paternal age [3].

Several studies have investigated the effects of paternal age on assisted reproduction outcomes. De la Rochebrochard reported that paternal age had a detrimental effect on pregnancy rates in conventional in vitro fertilization (IVF) cycles in cases of paternal and maternal ages >40 y and >35 y, respectively [4]. However, discrepancies exist when considering oocyte-donation cycles; while some studies indicated a negative influence on embryo quality, implantation rate and live birth rate [5–8], others suggested no effects on the rates of pregnancy, miscarriage and live births [9–12].

Although the age of the oocyte can be controlled in oocyte-donation cycles, the age of recipients cannot. Uterine receptivity of older recipients usually declined due to gynecological diseases, such as leiomyomas. Furthermore, the interaction between paternal age and maternal age can’t be evaluated in oocyte donation cycles.

The sample sizes of the reports described are limited and the conclusions are inconsistent. With nearly 10,000 IVF cycles included in the present study, we aimed to investigate the influence of paternal age on the reproductive outcomes in IVF cycles in a population of Chinese men.

## Materials and Methods

### Study population

This study is a retrospective analysis of IVF cycles performed at the Reproductive Medicine Center of the Third Affiliated Hospital of Guangzhou Medical University (China) between January 2007 and October 2013. The inclusion criteria were: 1. IVF cycles with embryo transfer between January 2007 and October 2013; 2. Agonist for pituitary downregulation; 3. Female age <39 y; 4. Retrieved oocytes >4; 5. Fresh embryo transfer; 6. Embryo transfer performed on Day 2 to Day 3.

The study was approved by the Ethics Committee of the Third Affiliated Hospital of Guangzhou Medical University. This clinical investigation was conducted according to the principles of the Declaration of Helsinki. Written informed consent was obtained from all participants.

### Methods

**Fig 1:**
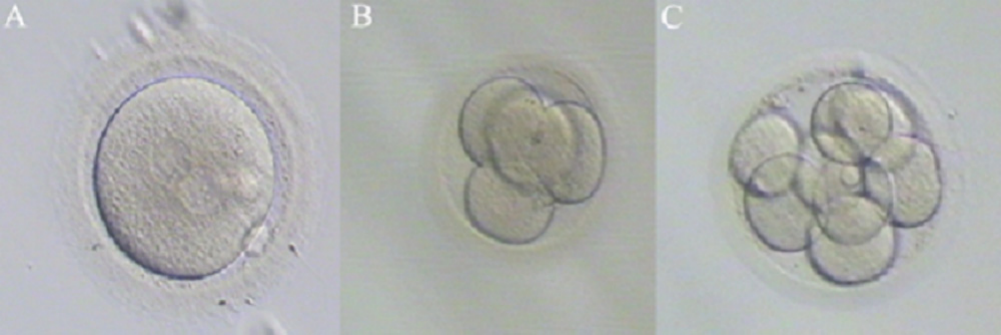
Images of embryos. 2PN on Day 1, 4-cell embryo on Day 2, 8-cell embryo on Day 3. PN = pronucleus.

Samples were grouped according to maternal age (<30, 30–34, 35–38) and then subdivided according to paternal age (<30, 30–32, 33–35, 36–38, 39–41, and ≥42 y). The main outcomes were embryo quality, and rates of fertilization, implantation, pregnancy, and miscarriage. High-quality embryos were defined as follows: embryos on Day 2 had 4–6 symmetric cells with ≤20% embryonic fragmentation (Fig 1B); embryos on Day 3 had 7–9 symmetric cells with ≤20% embryonic fragmentation (Fig 1C).

### IVF protocol

The gonadotropin-releasing hormone (GnRH) agonist (Triptorelin, Ipsen Pharma Biotech, France) 1.0–1.3 mg was administered in the luteal phase of the previous cycle. Tests of serum estrodial (E_2_), (luteinizing hormone (LH), follicule-stimulation hormone(FSH) and ultrasound were performed after 14 days of downregulation. Ovarian stimulation was started once the downregulation was satisfactory, i.e. serum E_2_ was <50 pg/ml, follicular diameter was 4–6 mm and endometrium thickness was <5 mm. When at least three follicles were ≥17 mm in diameter, ovulation was triggered by the administration of HCG. Oocytes were retrieved 34–36 h later and embryos were transferred 2 or 3 days after oocyte retrieval. Serum β-HCG was tested 14 days after transfer and ultrasound was performed 28 days after transfer if the β-HCG test was positive.

### Statistical analysis

One-way analysis of variance (ANOVA) was used for comparisons of means and comparisons of the rates of fertilization, implantation, pregnancy and miscarriage were analyzed by Chi-squared tests. Statistical analysis was performed using SPSS 21.0 software and *P* <0.05 was considered to indicate statistical significance.

## Results

**Fig 2:**
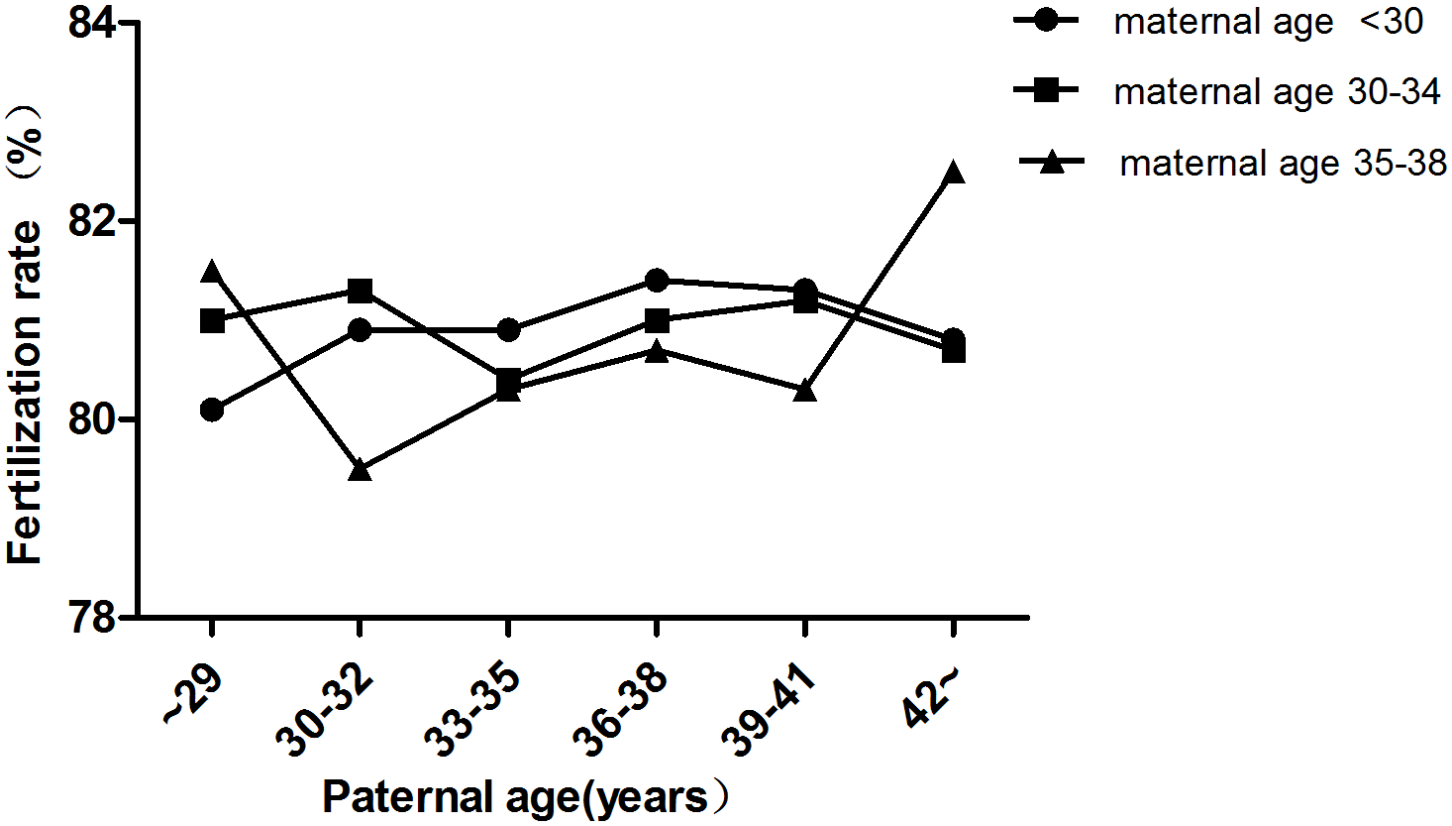
Fertilization rates in different age groups. In each group (maternal age <30 y, 30–34 y, 35–38 y), there were no significant differences in fertilization rate among different paternal age subgroups (*P*>0.05).

**Fig 3:**
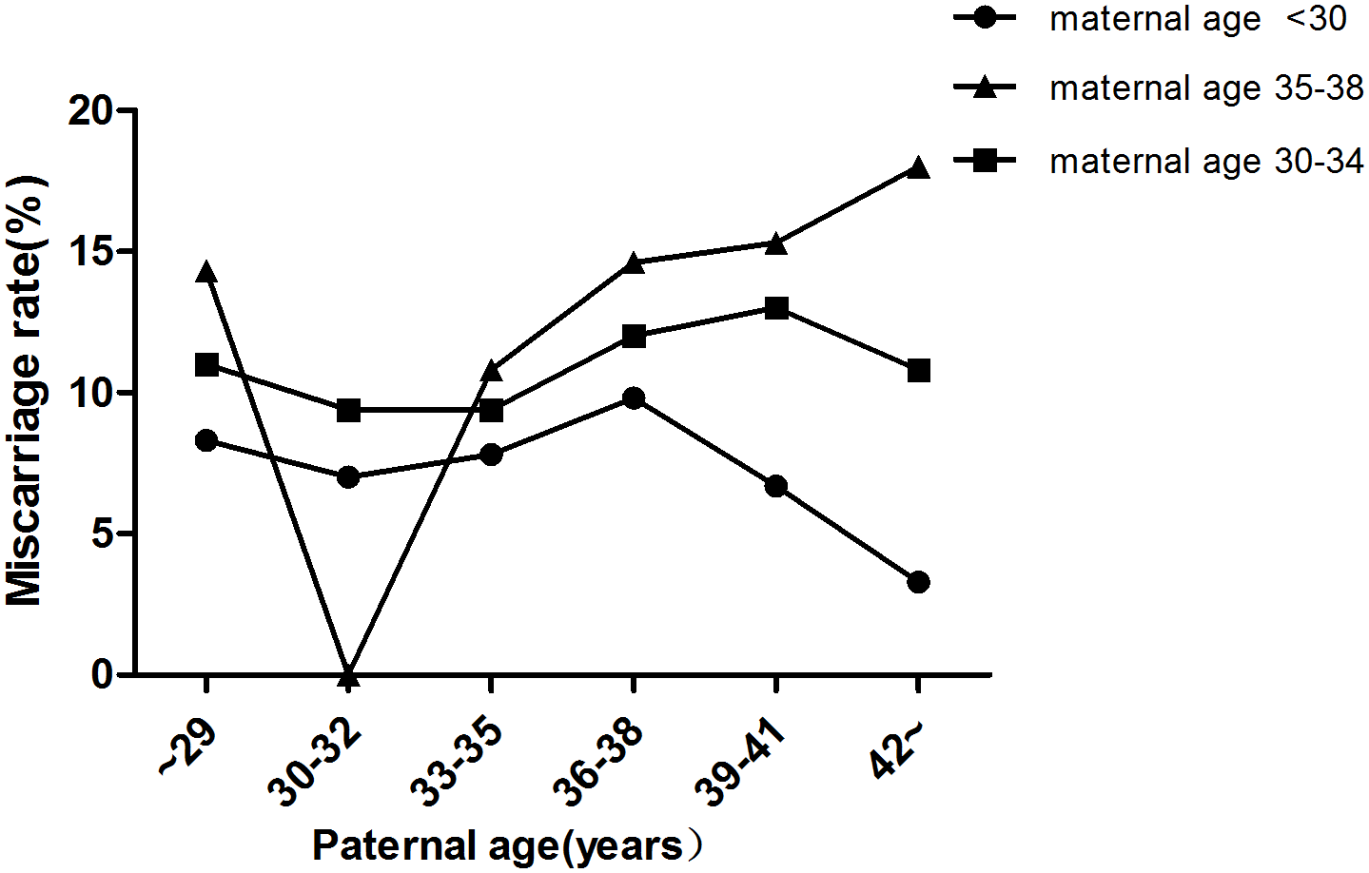
Miscarriage rates in different age groups. In each group (maternal age <30 y, 30–34 y, 35–38 y), there were no significant differences in miscarriage rate among different paternal age subgroups (*P*>0.05).

**Fig 4:**
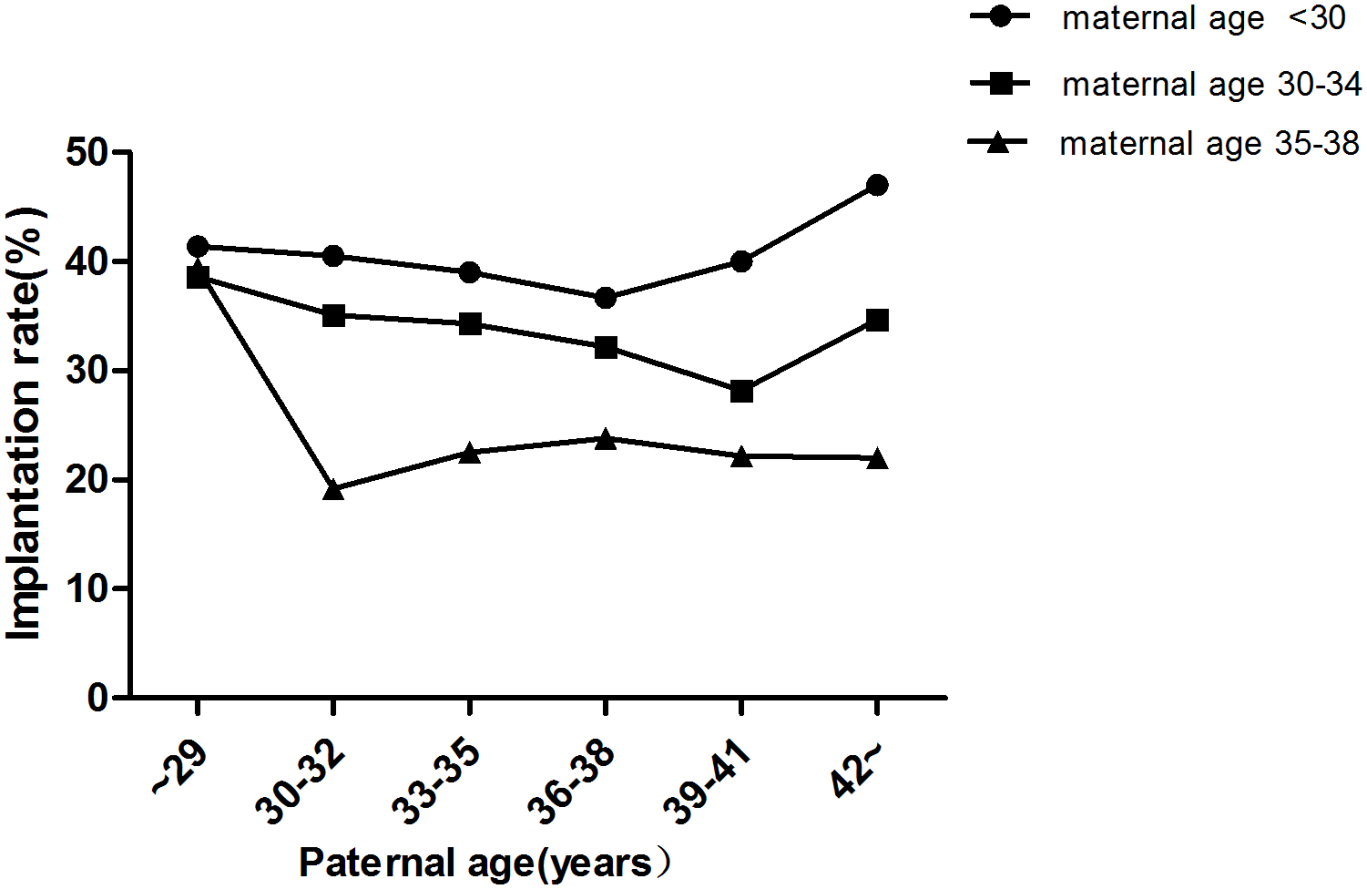
Implantation rates in different age groups. There were no significant differences in implantation rate among the different paternal age groups when the maternal age was <30 y and 35–38 (*P* >0.05). In the 30–34 y maternal age group, the implantation rates decreased with increased paternal age (*P* <0.05).

**Fig 5:**
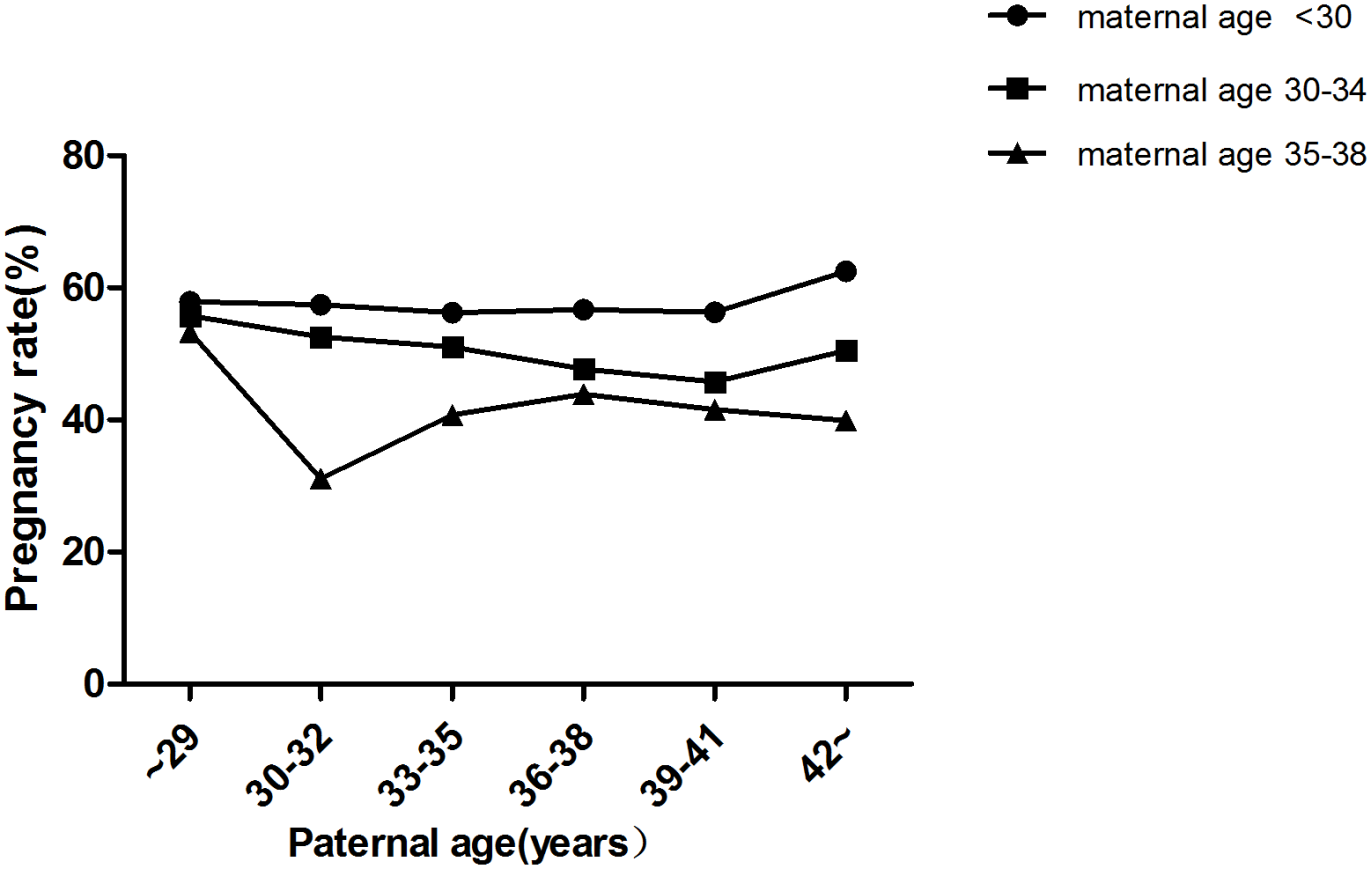
Pregnancy rates in different age groups. There were no significant differences in pregnancy rate among the different paternal age groups when the maternal age was <30 y and 35–38 (*P* >0.05). In the 30–34 y maternal age group, the pregnancy rates significantly decreased with increasing paternal age (*P* <0.05).

In total, 9,991 cycles were included in this study. Maternal age was <30 y in 3,327 cycles, 30–34 y in 4,587 cycles and 35–38 y in 2,077 cycles (Table 1). In each subgroup (maternal age <30 y, 30–34 y,35–38 y), there were no significant differences in terms of the fertilization rate (Fig 2), number of viable embryos (Table 2), number of high-quality embryos (Table 3) and miscarriage rate (Fig 3) among different paternal age groups (*P* >0.05). Chi-squared analysis revealed that there were no significant differences in implantation rate and pregnancy rate among the different paternal age groups when the maternal age was <30 y and 35–38 (*P* >0.05) (Figs 4 and 5). However, in the 30–34 y maternal age group, the implantation rate decreased with increased paternal age (38.6%, 35.1%, 34.3%, 32.2%, 28.2%, 34.7% in the <30, 30–32, 33–35, 36–38, 39–41 and ≥42 y paternal age groups, respectively; *P*<0.05) (Fig 4). In the 30–34 y maternal age group, the pregnancy rate was significantly higher in the <30 y (55.8%) and 30–32 y (52.6%) paternal age subgroups, compared with that in the 36–38 y (47.7%) and 39–41 y (45.8%) paternal age subgroups (*P* <0.05) (Fig 5).

**Table 1:** Cycle distribution in the different age groups.

Maternal age			Paternal age (years)				
(years)	<30	30–32	33–35	36–38	39–41	≥42	Total
<30	1443	1062	479	215	80	48	3327
	43.4	31.9	14.4	6.5	2.4	1.4	100
30–34	278	1241	1641	920	321	186	4587
	6.1	31.9	14.4	20.1	7	4.1	100
35–38	30	107	366	768	524	282	2077
	1.4	5.2	17.6	37	25.2	13.6	100
Total	1751	2410	2486	1903	925	516	9991
	17.5	24.1	24.9	19	9.3	5.2	100

Note: The upper row in each group is the number (n); the lower row is the percentage (%).

**Table 2:** Number of viable embryos in different age groups.

Maternal age (years)			Paternal age (years)				
	<30	30–32	33–35	36–38	39–41	≥42	*P*
<30	6.2±3.8	6.3±3.8	6.2±3.7	6.2±3.4	6.1±3.5	6.6±3.8	*P* >0.05
30–34	5.8±3.5	5.8±3.6	5.7±3.5	5.9±3.7	5.7±3.8	5.6±3.7	*P* >0.05
35–38	5.2±2.6	5.2±3.3	5.2±3.2	5.2±3.1	5.1±3.2	5.0±3.2	*P* >0.05

Note: No. of viable embryos = transferred embryos + frozen embryos

**Table 3:** Number of high-quality embryos in different age groups.

Maternal age (years)			Paternal age (years)				
	<30	30–32	33–35	36–38	39–41	≥42	*P*
<30	3.2±2.7	3.2±2.7	3.1±2.7	3.1±2.7	3.3±2.6	2.9±2.8	P >0.05
30–34	3.0±2.5	2.9±2.6	2.9±2.6	3.0±2.6	2.8±2.6	2.7±2.5	*P* >0.05
35–38	2.6±2.1	2.6±2.2	2.5±2.4	2.6±2.2	2.5±2.3	2.5±2.2	*P* >0.05

## Discussion

The results of the present study showed that paternal age had no significant effect on embryo quality at the cleavage stage and miscarriage rate; however, in the 31–34 y maternal age group, paternal age had detrimental effects on implantation rate and pregnancy rate.

Although our study did not reveal any association between paternal age and fertilization rate, which is in accordance with previous studies [5, 7, 10–12], two previous studies demonstrated that paternal age had an adverse impact on fertilization rate. One study of 227 intra-cytoplasmic sperm injection (ICSI) cycles showed significantly lower fertilization rate in the >50 y paternal age group compared with that in the <50 y paternal age group (P < 0.0001; OR = 1.36, 95% CI = 1.19–1.55)[13]. Another study of 672 oocyte-donation cycles reported by Luna et.al showed that the fertilization rate declined with increasing paternal age. When categorized by type of fertilization (ICSI vs. IVF), there were no differences in fertilization rates between the <40 and 40–49 y paternal age groups, but fertilization rate in IVF cycles was noted to be significantly lower in the ≥50 y paternal age group [8]. The study by Luna et.al investigated mainly selective ICSI cycles, in which most of the patients had male infertility. Although this study suggested that male aging has a detrimental effect on fertilization rates only when the sperm parameters are abnormal, these data must be interpreted with caution due to the limited sample size in a case-control study.

In our study, only embryos transferred at the cleavage stage were included in the analysis. It was notable that there were no significant differences in the number of high-quality embryos and viable embryos among the groups. This is consistent with other studies demonstrating that paternal age has no association with the quality of embryos at the cleavage stage [5, 7, 13, 14]. However, paternal age may have a negative impact on the rate of blastocyst formation when paternal genomes are activated contemporaneously [7, 8].

Although several studies have evaluated the effect of paternal age on implantation rate, the results are contradictory. While Luna reported that paternal age ≥60 y negatively affected implantation rates [8], Ferreira’s study indicated that paternal age had an adverse influence in ICSI cycles only in cases of male oligospermia [14]. In contrast, three other studies [5, 7, 15], as well as our own, documented no association between paternal age and implantation rates.

Nine studies showed no effect of paternal age on pregnancy rate [5, 7–13, 15], while Ferreira et.al documented an inverse relationship in oligozoospermic men, with a 5% of decrease in the pregnancy rate as paternal age increased by one year [14]. In our study, the detrimental effect of paternal age on the pregnancy rate was observed only in the 30–34 y maternal age group. There are two possible reasons for this: First, the sample size of this group is the largest (4,587 cycles);Second, the sample size was relatively well-distributed in this group. As older men tend to have older partners, the sample sizes of the two extremes were small. There were only 48 cycles in the subgroup of maternal age <30 y with paternal age ≥42 y and 30 cycles in the subgroup of maternal age 35–38 y with paternal age <30 y.

Our study, together with others [5, 8, 9, 12, 14–16], showed no correlation between paternal age and miscarriage rate, with the exception of one study reported by Frattarelli [7], which demonstrated a significant increase in pregnancy loss as paternal age increased.

There are several advantages in our study: First, the sample size is the largest among all the reported studies. Second, only cycles in which the maternal age was <39 y and at least four oocytes were retrieved were included in this study. In our analysis of the effects of paternal age, the study population was divided into three subgroups according to maternal age in order to control the bias from maternal age. Third, all the cycles were autologous IVF cycles. Most of the previous studies investigated oocyte-donation cycles, in which the donor age was usually <35 y. This approach limits the potential to evaluate the effects resulting from the interaction between paternal age and advanced maternal age. Previous studies of natural conceptions demonstrated that paternal age has an adverse impact on reproductive outcomes, especially among older women. A retrospective study of 6,188 randomly selected women showed that paternal age >40 y is a significant risk factor for infertility, but only in women aged >35 y [17]. In an investigation of 3,174 natural conceptions, de la Rochebrochard found that paternal age did not affect the miscarriage rate among the 20–29 y maternal age group, while the rate increased among 30–34 y maternal age group when paternal age was ≥40 y (OR = 2.90) and also in the ≥35 y maternal age group across all the paternal age groups (OR 3.38–9.18)[18]. In addition, the age of the recipients was not controlled in many of the oocyte-donating cycles [5, 7, 8, 10, 12]. Whitcomb et.al reported that live birth rates declined with increasing paternal age; nevertheless, this association was greatly attenuated when adjusted for recipient age [11]. The decrease in live birth rates with increasing recipient age may be explained by the increased incidence of gynecological diseases, such as leiomyoma, which damage endometrial receptivity. However, another recent study of 4,887 oocyte-donation cycles demonstrated that there was no association between recipient age and reproductive outcomes [9].

## Conclusions

Our study showed that paternal age had no significant effect on embryo quality at the cleavage stage and miscarriage rate. However, in the 30–34 y maternal age group, the implantation rate was decreased with increased paternal age and the pregnancy rates were significantly higher in the <30 y and 30–32 y paternal age groups compared with those in the 36–38 and 39–41 y paternal age groups.
